# The issues and challenges of addressing the importation of unregistered pharmaceutical products in Malaysia

**DOI:** 10.1080/20523211.2025.2523939

**Published:** 2025-07-08

**Authors:** Lay Peng Lim, Mohammad Firdaus Bin Abdul Aziz

**Affiliations:** a Faculty of Law, Universiti Malaya, Kuala Lumpur, Malaysia; b Pharmacy Enforcement Branch, Sabah State Health Department, Ministry of Health, Kota Kinabalu, Malaysia

**Keywords:** Import, unregistered products, medicines, pharmaceutical products, pharmacy enforcement

## Abstract

**Background:**

Unregistered pharmaceutical products, which have not undergone evaluation by the national regulatory agency, pose significant risks to public health. The continuous influx of these unregistered products into Malaysia highlights the inadequacies within the current regulatory approach. Existing literatures show that there is a gap in knowledge regarding the challenges encountered by enforcement agencies during inspection of imported pharmaceutical products at entry points. Therefore, this study aims to identify the issues and challenges encountered by enforcement officers in curbing the importation of unregistered products and propose recommendations that can be considered to enhance legal enforcement.

**Methods:**

This study employed a qualitative approach by conducting one-to-one interviews with twelve Pharmacy Enforcement Officers and Customs Officers to gather relevant insights.

**Results:**

The key issues and challenges encountered in controlling the importation of unregistered products were categorised into several themes, including (i) organisational constraints; (ii) technical challenges; (iii) modus operandi employed by importer; and (iv) external challenges, such as proliferation of online platforms and persistent market demand for unregistered products.

**Conclusion:**

The findings revealed that the issues and challenges faced by enforcement officers stem from the inadequacies identified in the existing laws and regulatory practices. Multifaceted approaches that encompass legislative reforms, administrative transformation and rigorous enforcement are necessary in fostering more effective control over the importation of unregistered products in Malaysia.

## 1. Introduction

### 1.1. Background study

Medicines or pharmaceutical products play a fundamental role in the healthcare system, serving not only to combat illness but also to prevent diseases and promote general well-being. Worldwide, annual expenses allocated by governments for pharmaceutical products are growing exponentially, indicating the significant contribution of these products to improving patient health outcomes (World Health Organization, 2021). To ensure the safety, efficacy and quality of pharmaceutical products for human consumption, a robust legal and regulatory system is indispensable (Nyika et al., 2022). In Malaysia, all pharmaceutical products must be registered with the National Pharmaceutical Regulatory Agency (NPRA) before they can be imported, manufactured or marketed for sale (Nur Wahida et al., 2016a). The term ‘pharmaceutical products' in this context encompasses: (1) controlled medicines (also referred to as ‘Poisons' in accordance with Malaysian Poisons Act 1952); (2) over-the-counter (OTC) products; (3) traditional and homeopathic medicines; (4) natural products with therapeutic/modern claims; and (5) health supplements (National Pharmaceutical Regulatory Agency, 2025). Product registration process involves thorough testing, evaluation and assessment to ensure compliance with established quality standards. Registered pharmaceutical products in Malaysia can be visually identified by the presence of a registration number beginning with the prefix ‘MAL' and a FarmaTag™ security hologram label on the outer packaging (Pharmaceutical Services Programme, 2021a).

The World Health Organization (WHO) adopts the term ‘falsified  to describe counterfeit or fake medicinal products that are fraudulently misrepresented in term of their identity, composition or source (World Health Organization, 2024). In the Malaysian legal framework, there is no explicit definition for ‘falsified' or ‘counterfeit' pharmaceutical products. However, the Control of Drugs and Cosmetics Regulations 1984, enacted under the Sale of Drugs Act 1952, mandates registration of pharmaceutical products as outlined in Regulation 7(1)(a). Therefore, products that have not undergone this regulatory assessment and approval process are known as ‘unregistered' products within the Malaysian regulatory context (Nur Wahida et al., 2016b).

Unregistered products are those that have not undergone any assessment and evaluation by relevant authorities. Hence, there is a high possibility that unregistered products are counterfeit, adulterated with prohibited substances, contaminated, or even containing inadequate active ingredients (Ong et al., 2020). Consumption of these products could jeopardise public health. A study conducted in Malaysia on the adverse effects of traditional medicines showed that 70% of the deaths were caused by consumption of unregistered products (Sameerah Shaikh & Zoriah, 2020). Moreover, since unregistered products from foreign countries are manufactured in facilities that have not been inspected by relevant authorities, it is impossible to verify their compliance with good manufacturing practice as well as adherence to storage, labelling, and packaging requirements (Bro, 2005). For instance, Pullirsch et al. (2014) reported that high levels of microbial contaminations were detected in illegal medications manufactured in less hygienic conditions. Therefore, in real life setting, the quality and safety aspects of unregistered products cannot be assured.

Nevertheless, the pervasive issue of unregistered products remains a longstanding challenge. In the era of advanced technology, social media and e-commerce platforms have become direct conduits for the distribution of unregistered products (Marina et al., 2021). The convenience and wide selection offered by online platforms enable consumers to easily access various pharmaceutical products via the Internet, even from overseas. The recent COVID-19 pandemic witnessed a surge in demand for unregistered herbal supplements from China, such as ‘Lianhua Qingwen Jiaonang’, purportedly used to treat the viral infection (Nurhidayati, 2020). This scenario poses enforcement challenges particularly when the substantial influx of unregistered products into the local market is driven by extensive importation. Numerous reported cases of seizures and raids conducted in different states of Malaysia have revealed that many shipments of unregistered products have bypassed regulatory checks during importation (Audrey, 2021; Selangor Journal, 2021).

### 1.2. Existing regulatory framework in Malaysia

#### 1.2.1. Relevant laws and regulations

The importation of pharmaceutical products into Malaysia is primarily governed by the Control of Drugs and Cosmetics Regulations 1984. Meanwhile, pharmaceutical products containing scheduled poisons and dangerous drugs are subject to additional regulatory requirements under the Poisons Act 1952 and Dangerous Drugs Act 1952 along with their associated regulations including Poisons Regulations 1952, Poisons (Psychotropic Substances) Regulations 1989, and Dangerous Drugs Regulations 1952. As such, the provisions under Control of Drugs and Cosmetics Regulations 1984 must be read in conjunction with these laws to ensure compliance with all applicable regulations for lawful importation.

According to Regulation 7(1) of the Control of Drugs and Cosmetics Regulations 1984, pharmaceutical products must be registered, and only importers with a valid import licence issued by the Director of Pharmaceutical Services under Regulation 12(1)(d) of the Control of Drugs and Cosmetics Regulations 1984 are permitted to import them. Notwithstanding, exemptions exist within the laws that allow importation of unregistered products without the need for import licences such as government officials importing products as part of their official duties, and individuals exempted by the Minister. Regulation 7(3) of the Control of Drugs and Cosmetics Regulations 1984 exempts individuals who import products as part of their luggage solely for personal or family use, provided that the quantity doesn’t exceed the amount reasonably required for one person’s one-month usage. Furthermore, Regulation 15(6) of the Control of Drugs and Cosmetics Regulations 1984 gives leniency for the importation of unregistered products solely for treatment of life-threatening illnesses with pre-approval from the Director of Pharmaceutical Services, primarily caters to hospital and healthcare institutions in facilitating procurement of unregistered medications for their patients.

The Poisons Act 1952, a statute that regulates the import, export, manufacture, and sale of prescription and non-prescription medicines, spells out in Section 8 that only individuals possessing a valid licence under Section 26 are authorised to import poisons from outside Malaysia. Section 8(2) of the Poisons Act 1952 stipulates that the requirements for import licence is not applicable for individuals bringing in a one-month supply of poisons for personal use via luggage, letter or parcel post, government officials as part of their duties, and any person exempted by the Minister. Additionally, Regulation 4 of the Poisons Regulations^1952^ outlines specific requirements that must be fulfilled for importation of poisons by post, including clear labelling on the package with the patient’s name, name of the medicines, quantity supplied, and date of posting to avoid seizure by enforcement officers.

For prescription medicines containing psychotropic substances as listed in the Third Schedule of the Poisons Act 1952, import authorisations are required in accordance with Regulation 4(1) of the Poisons (Psychotropic Substances) Regulations 1989. However, importation of a one-month supply of psychotropic substances for personal use via luggage is exempted from the authorisation requirements if accompanied by a valid prescription from a qualified medical practitioner, as stated in Regulation 4(2). Meanwhile, importation of pharmaceutical products containing dangerous drugs requires import authorisation as stipulated in Section 20 of the Dangerous Drugs Act 1952. Section 25 outlines exemptions for travellers entering Malaysia who bring in reasonable quantities of dangerous drugs for personal use, provided they possess a valid prescription from a medical practitioner issued in accordance with the legal requirements of the country where the drugs were procured, and declare the medications to the authorities upon arrival in Malaysia.

In sum, the provisions within Malaysian existing law, specifically Control of Drugs and Cosmetics Regulations 1984, unequivocally prohibit importation of unregistered pharmaceutical products for commercial purposes. Nevertheless, confusion may arise from the exemptions granted for importing distinct pharmaceutical categories for personal consumption, as there exists a lack of synchronisation among the three Acts involved – Sale of Drugs Act 1952; Poisons Act 1952, and Dangerous Drugs Act 1952 – in relation to such exemptions. Inconsistencies in the imposed restrictions may pose challenges in achieving compliance, particularly when our laws are not fully observed by the public. Table 1 summarises the importation requirements for different product categories and personal use exemption.

**Table 1.** Overview of importation requirements for different product categories and personal use exemption.

Product categories	**Non-poisons (OTC, traditional/natural products, health supplements)**	**Prescription medicines containing scheduled poisons**	**Prescription medicines containing psychotropic substances**	**Prescription medicines containing dangerous drugs**
Legislation governing the importation	SODA 1952 and its regulations	SODA 1952 and its regulations Poisons Act 1952 and its regulations	SODA 1952 and its regulations Poisons Act 1952 and its regulations	SODA 1952 and its regulations Poisons Act 1952 and its regulations DDA 1952 and its regulations
Importation requirements: a) Must be registered products	r. 7(1)(a) of the CDCR 1984	r. 7(1)(a) of the CDCR 1984	r. 7(1)(a) of the CDCR 1984	r. 7(1)(a) of the CDCR 1984
b) Types of import licence or authorisation	1. Import licence under CDCR 1984	1. Import licence under CDCR 1984 2. Type A Licence under Poisons Act 1952	1. Import licence under CDCR 1984 2. Type A Licence under Poisons Act 1952 3. Import authorisation under P(PS)R 1989	1. Import licence under CDCR 1984 2. Type A Licence under Poisons Act 1952 3. Import authorisation under DDA 1952
Provisions applied for personal use exemption	r. 7(3) of the CDCR 1984	s. 8(2) of the Poisons Act 1952 and r. 4 of the Poisons Regulations 1952 r. 7(3) of the CDCR 1984	s. 8(2) of the Poisons Act 1952 and r. 4(2)(a) of the P(PS)R 1989 r. 7(3) of the CDCR 1984	s. 25 of the DDA 1952 r. 7(3) of the CDCR 1984
Restrictions for personal use exemption under the provisions: a) Product registration status	Exempted from registration	Exempted from registration	Exempted from registration	Exempted from registration
b) Quantity allowed	One month supply for one person usage	One month supply for one person usage	One month supply for one person usage	Not specified under s. 25 of the DDA 1952
c) Import for family member	Allowed	Allowed	Allowed	Not allowed under s. 25 of the DDA 1952
d) Prescription by medical practitioner	Not required	Required	Required	Required
e) Method of importation	Baggage	Baggage, letter or parcel post	Baggage	Baggage
f) Labelling requirement	Not specified	Only mentioned for import via letter or parcel post	Not specified under r. 4(2)(a) of the P(PS)R 1989	Not specified under s. 25 of the DDA 1952

Note: CDCR 1984 = Control of Drugs and Cosmetics Regulations 1984; DDA 1952 = Dangerous Drugs Act 1952; P(PS)R 1989 = Poisons (Psychotropic Substances) Regulations 1989; SODA 1952 = Sale of Drugs Act 1952.

#### 1.2.2. Regulatory bodies

**Figure 1.**
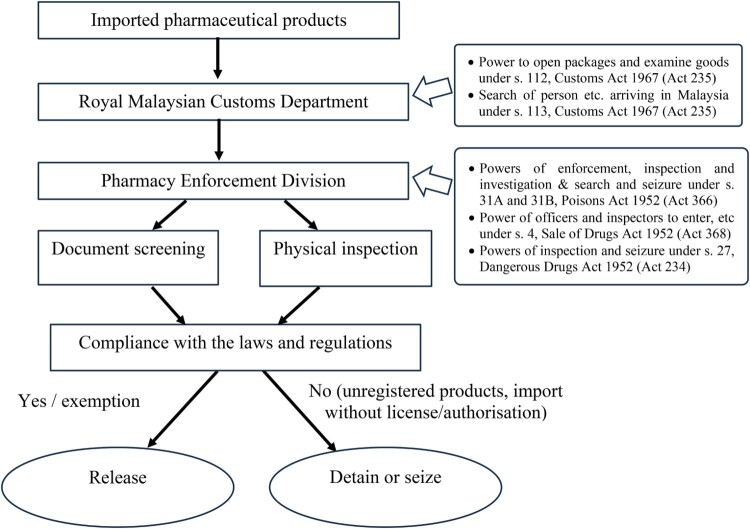
Flowchart of inspection process for pharmaceutical products at entry points.

The Pharmacy Enforcement Division (PED) under the Ministry of Health Malaysia (MOH) is responsible for enforcing forementioned laws and regulations related to pharmaceutical products (Paraidathathu, 2020). This agency collaborates with the Royal Malaysian Customs Department (RMCD) at entry points such as airports, ports, and mail and courier hubs to control the importation of pharmaceutical products via land, air, and sea. When Customs Officers encounter imported goods that require clearance from Other Government Agencies (OGA), for instance pharmaceutical products declared under Harmonised System (HS) Code overseen by PED, they refer such commodities to Pharmacy Enforcement Officers (PEOs) for technical advice before granting entry into the country (Ting et al., 2017). PEOs would meticulously scrutinise documents submitted by importers, including proof of product registration, import licence or authorisation, and product information such as labels, leaflets, or pictures. Physical inspections will be conducted when deemed necessary (Pharmaceutical Services Programme, 2020, Jun). Upon determining that the imported products comply with the laws and regulations enforced by PED, PEOs would have no objection to releasing the goods to the importers. If any unlawful importation of pharmaceutical products is identified, PEOs will provide advice to the Customs Officers for the detention or seizure of such items to prevent their entry into the country (Ting et al., 2017). Figure 1 illustrates the inspection process for pharmaceutical products at entry points.

In accordance with Section 3 of the Sale of Drugs Act 1952, PEOs are appointed as analysts, officers, and inspectors. Hence, offences related to importation of unregistered products may be subject to investigation and prosecution by PED. Moreover, as a preventive approach to curb illicit importation of unregistered products, PED has initiated website surveillance activities through collaborative efforts with the Malaysian Communications and Multimedia Commission (MCMC), Malaysia Network Information Centre (MYNIC) and platform providers, with the aim to crackdown on illicit webpages offering unregistered products (Pharmaceutical Services Programme, 2021b). PED has also taken the lead in conducting the ‘Online Drug Purchase Awareness Campaign’ aimed at raising awareness about the hazards of purchasing unregistered products on the internet since 2015 and actively involved in Operation Pangea since 2013, an international operation led by the INTERPOL to combat illicit pharmaceutical trade, demonstrating its commitment to halting the influx of unregistered products into Malaysia (Pharmaceutical Services Programme, 2019). However, the reported proliferation of unregistered products suggests that the existing legal and regulatory system may still be inadequate in combating the importation of unregistered products.

### 1.3. Identified challenges to existing regulation

A few local studies have pointed out challenges in addressing the importation of unregistered products. Haniff Mohd Nawi et al. (2020) suggested that the higher prevalence of unregistered product use in the state of Kelantan could be attributed to its close proximity to Thailand, rendering the state more susceptible to cross-border illegal activities, including trafficking of medicines, thus facilitates easy access to unregistered products originated from a neighbouring country. Ting et al. (2017) also expressed a similar issue, where the lack of manpower and logistical resources to station PED officers at nine borders between the state of Sarawak in East Malaysia and the state of Kalimantan in Indonesia could provide opportunities for smugglers to illicitly transport counterfeit products from Indonesia into Malaysia without detection by law enforcement authorities. In their study on pharmacists’ perception of laws and regulations towards unregistered products, Nur Wahida Zulkifli et al. (2016b) discovered that while a majority of the respondents expressed the view that the existing laws are capable of effectively managing unregistered drugs in Malaysia, the effectiveness of these laws and regulations has been compromised by certain inefficiencies of its enforcement. Specifically, shortage in law enforcement and limited public awareness have contributed to the proliferation of unregistered products. These studies revealed a gap in knowledge regarding the challenges encountered by enforcement agencies during inspection of imported pharmaceutical products at entry points, as regulatory processes are not commonly published.

Hence, this study aims to identify key challenges and issues that are encountered by enforcement officers in combating importation of unregistered pharmaceutical products. Ultimately, this study offers recommendations on how the current legislation and regulatory approaches can be improved to strengthen control over the importation of unregistered products into Malaysia.

## 2. Methods

Qualitative interviews were conducted to obtain first-hand insights from key stakeholders namely experienced officers from PED and RMCD. A purposive sampling technique was used to select interviewees based on specific criteria. Officers who have been stationed at international entry points, including airports, ports, postal and parcel service hubs, with a minimum of 2 years of working experience were chosen. Additionally, senior officers from the PED who possessed exclusive knowledge and have been actively involved in website surveillance, investigation, or prosecution of cases relating to unlawful importation of pharmaceutical products were also recruited.

The determination of sample size for this study was rooted in the concept of data saturation. Guest et al. (2006) recommended a minimum of twelve participants for qualitative studies to achieve saturation while Hennink and Kaiser (2022) concluded that saturation could be achieved with nine to seventeen interviewees. Based on this guidance as well as due to data saturation was achieved after interviewing twelve participants, the data collected was therefore, deemed adequate for this study, and the interview process was then concluded.

The participants comprised ten interviewees from PED and two interviewees from RMCD. A greater number of participants were selected from PED as it is the primary authority responsible for enforcing pharmaceutical laws at Malaysia’s entry points and thus it is believed that their in-depth operational knowledge and practical field experience positioned them as key informants for examining the enforcement challenges associated with the importation of unregistered pharmaceutical products. Meanwhile, the inclusion of two RMCD officers served to complement the PED’s perspectives by providing insights into the coordination between enforcement agencies at the border.

Before the commencement of the interviews, ethical approval for this study was first obtained from two research ethics committees namely the Universiti Malaya Research Ethics Committee (UM.TNC2/UMREC_2402) and Medical Research & Ethics Committee under the MOH (NMRR-ID-23-00415-YYY). Subsequently, formal permission to access the study sites was sought from the respective Heads of Department of the PED and RMCD. All the twelve interviewees were recruited via formal email invitations. Between May and June 2023, in-person interviews were conducted with eleven interviewees located in the Klang Valley while an online interview was conducted with one interviewee residing outside of the Klang Valley. The selected interviewees were provided with a participant information sheet and a consent form. Interviewees who willingly agreed to partake in the study were required to sign the consent form prior to attending the interview sessions.

The semi-structured interview guide employed during the interviews was developed through a dual approach. It was initially designed based on a comprehensive review of relevant literature and subsequently refined through feedback from experienced researchers in the relevant field. Interview sessions were recorded with the participants’ consent and transcribed verbatim by the first author. Thematic analysis, guided by Braun and Clarke’s framework, was employed to analyse the collected data to identify recurring themes and patterns within the interviewees’ perspectives on the issues and challenges pertaining to the study (Braun & Clarke, 2012). The analysis began with data familiarisation through repeated readings of the interview transcripts, followed by the generation and labelling of codes before the codes were subsequently organised, reviewed, and refined into themes and subthemes as presented in the study findings.

## 3. Results

The issues and challenges identified from the interviews were grouped into several themes: (i) organisational constraints, (ii) technical challenges, (iii) modus operandi by the importers, and (iv) external challenges (Table 2).

**Table 2.** Categorisation of themes.

**Main themes**	**Sub-themes**
Organisational constraints	Lack of authority at entry points
	Manpower and equipment limitations
	Lack of enforcement power, investigative power and experience
Technical challenges	Difficulties in establishing importation
	Issues in product classification
	Improper labelling and labelling in foreign languages
Modus operandi by the importers	Deliberate concealment of recipient details
	False declaration of goods and document falsification
External challenges	Proliferation of websites and online platforms
	Market demand

### 3.1. Theme 1: organisational constraints

#### 3.1.1. Sub-theme 1: lack of authority at entry points

Most respondents voiced out that the responsibility for controlling imports primarily lies with Customs Officers, while PEOs serve merely as technical advisors.

*“The problem is we only received referrals from Customs. We are not the first line there who can confirm the types of items chosen to be scanned … Without the referral, there will be no consignment to be checked by our side.”* (Interviewee 1)

*“The Customs control the entry point … In terms of scanning, it's not up to us to decide.”* (Interviewee 6)

*“As a PED, we do not have specific things to filter all of the parcels that come in here, we must rely on the Customs.”* (Interviewee 2)

#### 3.1.2. Sub-theme 2: manpower and equipment limitations

Some of the interviewees also raised issue regarding shortages of manpower and insufficiency of cargo scanners at entry points, which affects the efficiency of enforcement. There was an attempt initiated by enforcement officers to collaborate with cargo terminal operators (CTOs) to utilise their scanners, however, such an initiative was to no avail due to cost issue.

*“The shortage of custom officers in entry point causes low numbers of parcels being scanned everyday … Another problem is the shortage of scanners … Eventually it affected the number of consignments referred to PED.”* (Interviewee 1)

*“Last time we tried to make a potential collaboration with them (CTOs) … They asked, ‘Who wants to bear the cost?’ … so the deal didn’t happen.”* (Interviewee 4)

#### 3.1.3. Sub-theme 3: lack of enforcement power, investigative power and experience

The interviewees also shared that their investigative power is restricted and unlike their counterparts in the police force, who are also mandated with investigative power, they argued that they are not equipped with necessary power such as handling a gun if they find themselves in an unwanted situation. PEOs are professionally trained as pharmacists, and thus they have limited legal knowledge, skills and experience in carrying out investigation as well as dealing with suspects.

*“The regulation (Control of Drugs and Cosmetics Regulations* 1984*)* *doesn't even talk about power. When we* *want to investigate, sometimes we have power restrictions to investigate … It has to be comprehensive, just like the (amended) Poisons Act, (where) our power is like the police power under the Criminal Procedure Code. Power to access premise, for seizure, to issue a notice to the person present to give information, to access any information … ”* (Interviewee 5)

*“Our investigating officer is totally different from the police. We do not hold any guns. We are not being trained if there's any havoc caused by the suspects … We do not have power to detain or catch a person as a suspect.”* (Interviewee 2)

*“Sometimes we have a lead, but all of the witnesses are in the dangerous or black area such as Pulau Sebatik, so the investigating officer doesn’t have experience to go into the black area and identify the person.”* (Interviewee 2)

*“Sale of Drugs Act 1952 should also have compounds to facilitate our enforcement activities.”* (Interviewee 5)

### 3.2. Theme 2: technical challenges

#### 3.2.1. Sub-theme 1: difficulties in establishing importation

A lack of clear statutory definition of ‘import’ – as to what constitutes as imported pharmaceutical products, was highlighted by interviewees as a significant problem in carrying out their duties. This is particularly challenging if the product is claimed to be on transit. Also, the interviewees highlighted that they are aware of a loophole in the regulatory measures, whereby there are some places where smugglers can rathole unregistered products into the country because there is no customs available at these places.

*“If you look at our law (Sale of Drugs Act* 1952*)* *currently, there is no clear definition about import.”* (Interviewee 7)

*“We have problems if the case is a transit case because the goods have not yet reached the importer. So, you don't know who the final importer is. The only entity who can verify the importer is the exporter. But to get cooperation from the exporter, issue number one, exporter is not in the country. Number two, the exporter may refuse to give statement. If you don't get all that (evidence), how do you want to proceed?”* (Interviewee 7)

*“ … Those illegal holes, where smugglers just bring in, there is no custom checkpoint. That is even more difficult because we don't have any documentation (for declaration of imported goods).”* (Interviewee 12)

#### 3.2.2. Sub-theme 2: issues in product classification

Another challenge that the interviewees are facing is the technical knowledge in identifying pharmaceutical products classification, which does not fall within their expertise. In doing so, they need the assistance from officers from the National Pharmaceutical Regulatory Agency (NPRA). They will need to refer the case to NPRA, and it was argued that such a process takes time and will incur higher cost to the parties at entry points.

*“When it comes to product classification, the entity who is supposed to determine whether a product needs to be registered or not is not the PEO, it's the DCA. But DCA or the Secretariat (NPRA), they are not at the entry point. At entry point, things are moving fast. And each time involves dollars and cents. You need to make a fast decision … We have problems if the thing requires other parties’ decision … ” (Interviewee 7)*

*“We (PEOs) don't do the classification. That's not our job scope.”* (Interviewee 9)

#### 3.2.3. Sub-theme 3: improper labelling and labelling in foreign languages

Some interviewees faced with uncertainties that are caused by improper labelling such as incomplete name, labels are separated from the products resulting in the difficulty to determine the right labels, and labels that are written in foreign languages other than English.

*“ … The product presentation is doubtful. For example, the name is not complete, there is no label, no complete label … ”* (Interviewee 8)

*“There's a case where the label is separated from the product, and the product itself contains a pharmaceutical dosage form. But we can't manage to prove that the label is for this product because the label is separated.”* (Interviewee 2)

*“Imported products from countries use different languages other than Bahasa Malaysia and English such as Japan, Korea … So the labelling and also the language on the labels have caused us problems in making decisions.”* (Interviewee 1)

### 3.3. Theme 3: modus operandi by the importers

#### 3.3.1. Sub-theme 1: deliberate concealment of recipient details

The interviewees expressed that some illicit importers deliberately conceal their true identities by employing tactics such as using fake names and addresses to import unregistered products. Transportation of unregistered products into Malaysia by individuals without identity documents adds complexity to law enforcement efforts to identify the primary suspect.

*“The name is like John Doe. It could be anyone and (anyone’s) phone number on the parcel.”* (Interviewee 2)

*“The receivers of these unregistered or illegal products, they didn't put their actual names. They didn't put their actual premises in the recipient details.”* (Interviewee 1)

#### 3.3.2. Sub-theme 2: false declaration of goods and document falsification

One interviewee pointed out concerns surrounding false declarations of goods and document falsification. Specifically, some importers or agents intentionally misclassify pharmaceutical products as other types of commodities to circumvent inspections by relevant agencies and sidestep the perceived red tape involved in importation.

*“ … Usually, they will declare the parcel as ‘documents’, or ‘box’ … If we see ‘documents’ and the importer is not an embassy or a company, we can just directly ask for an inspection.”* (Interviewee 4)

*“(Importers) do false documentation on the airway bill … How do we know? Because documents look almost the same. Sometimes we do random checking, just to see if there's any pattern.”* (Interviewee 4)

*“For the importation side, the agent should be more transparent.”* (Interviewee 3)

### 3.4. Theme 4: external challenges

#### 3.4.1. Sub-theme 1: proliferation of websites and online platforms

Some of the interviewees believe that technological advancements, particularly the Internet, significantly facilitate the importation of unregistered products into our country. The inadequate capability to detect and effectively control the availability of these products across various online platforms has led to rampant influx of unregistered products.

*“If you go to whatever (platform), everything is easily obtained, with just a single click … It is a burden to us in terms of monitoring it … ”* (Interviewee 7)

*“The main factor in contributing to high volume of importation and pooling of the unregistered products is online sales that are booming so fast right now. In these e-platforms, they offer way cheaper price compared to those products that are available in our local market.”* (Interviewee 1)

*“Even though we monitor or take down a lot of websites, but still the number of websites available is a lot. You take down ten in the morning, in the afternoon it will double up to twenty or more.”* (Interviewee 6)

*“In terms of blocking, I think it should be in the law … At least we have the power to request or to report to the platform for them to remove the content or to block access to the websites … The problem is, our legal advisor said that if we put that power in our law, it's as if we are overruling the power of the MCMC. But I don't see it that way because we only do our job, which is related to the pharmaceutical products. If MCMC put that power in their Act, they also have a problem because they don't have any medical background to make decision … ”* (Interviewee 6)

#### 3.4.2. Sub-theme 2: market demand

Most interviewees contend that the demand from local consumers is a driving factor behind the influx of unregistered products. This demand is influenced by perceived suitability and higher quality of unregistered products manufactured in developed nations but unavailable in the local market. The COVID-19 pandemic has also witnessed a significant surge in demand for Ivermectin and traditional products from China, many of which were dubiously promoted as treatments or preventive measures for COVID-19 despite a lack of scientific evidence. Additionally, preferences among foreigners and lower product costs further fuel the demand for unregistered products from overseas.

*“The people who use these unregistered products-somehow I still believe that Malaysians believe the outsiders can make better medicines than us.”* (Interviewee 3)

*“Usually, demand. Sometimes, rumours play a role. During the pandemic, there was a medicine (Ivermectin) claimed for COVID. So, the increase in purchase and entry of the medicine is obvious.”* (Interviewee 11)

*“People want the (unregistered) product to become fair and beautiful, even though they know it's dangerous.”* (Interviewee 10)

*“We have a lot of foreigners here who actually trust their home country's product more than the local products here.”* (Interviewee 12)

*“The demand is there. For example, the sex medicine Viagra. From India, they have this Kamagra. It's cheaper than ours, than what we sell here.”* (Interviewee 4)

## 4. Discussion

The dependency on referrals from Customs Officers underscores the fact that PED lacks complete control over inspection processes. Given the current system, one could argue on the effectiveness of the scanning process of imported pharmaceutical products solely carried out by Custom Officers as to whether or not they are able to do it properly. A local study found that Customs Officers with less than ten years of working experience exhibit significantly lower awareness on counterfeit pharmaceutical products compared to those with over a decade’s experience (Ting et al., 2017). This discrepancy is attributed to the inherent absence of medical backgrounds among Customs Officers, who primarily gain experience through practical exposure. Consequently, their constrained capability to discern and detect pharmaceutical products necessitating referral to PED may enable unregistered products to evade scrutiny. Recognising that curbing importation of unregistered products cannot be achieved in isolation, this paper therefore argues that fostering collaboration between PED and other enforcement agencies should become imperative to exert maximal control over imported unregistered products in Malaysia.

Manpower and equipment limitations have forced the enforcement officers to only prioritise their inspections on high-risk shipments. Based on this account, it can be argued that not only the enforcement and regulatory measures are ill-equipped with necessary infrastructure, which can be a stumbling block in ensuring inspection can be appropriately carried out by the officers, but also there seems to be a financial constraint. It is crucial for the relevant authorities or agencies in the government to take on board that currently, there is insufficient resources to ensure comprehensive inspection of all imported pharmaceutical products, which suggest a lack of enforcement and this finding is in line with the finding reported in the study by Nur Wahida Zulkifli et al. (2016b) that there is a lack of enforcement involving unregistered drugs despite the regulations are viewed as comprehensive by some enforcers. Therefore, to facilitate enforcement officers to be more efficient in carrying out their inspection, it is vital for the government to invest in necessary technology and allocate adequate funding for the enforcement purposes.

The lack of enforcement power, investigative power and experience, as shared by the interviewees, hampers the PEOs from conducting a robust investigation (Ruzita et al., 2009). In contrast to Nur Wahida Zulkifli et al. (2016b) that attributes shortage of law implementation as a pivotal factor, this study brings to light the coexistence of inadequacies within the existing laws that contribute to inefficiencies in curbing unregistered products. Particularly, the limited authority vested in PEOs under Sale of Drugs Act 1952, where there are no specific provisions to detain suspects or witnesses, or request them to provide statements, further exacerbates challenges in investigating importation of unregistered products. Another lacuna lies in the absence of provisions empowering PEOs to exercise the option of compounding offences as alternatives to prosecutions. Legislative overhaul in Malaysia is necessary to enhance overall efficiency in combating importation of unregistered products.

Our finding aligns to those reported by Haniff Mohd Nawi et al. (2020) and Ting et al. (2017), who highlighted the vulnerabilities of certain regions in our country to cross-border illegal importation due to geographical proximity and inadequate enforcement resources. The criminal conduct demonstrated by these importers can be explained through Routine Activity Theory developed by Cohen and Felson (1979), where the absence of capable guardians or enforcers at the borders inevitably creates opportunities for offenders to engage in illicit activities, including the smuggling of unregistered products. In essence, the lack of effective oversight allows offenders to exploit the perceived loopholes in enforcement measures, operating with the assumption that their chances of being detected and apprehended are low. This scenario underscores the critical imperative for enhanced enforcement efforts to effectively combat such transgressions.

Our findings also show the challenge that PEDs would face in real life especially if they are uncertain about the accurate classification of imported items referred by Custom Officers for inspection – whether they constitute pharmaceutical products falling under their jurisdiction or non-pharmaceutical products that are governed by other health departments. As the authority responsible for product classification lies with the NPRA, their absence at entry points further complicates the decision-making process. It is essential to accurately determine products classification either as medicines, food, or fall into an ‘in-between’ category to ascertain compliance with relevant laws and regulations, considering different category is subject to distinct regulatory frameworks (Da Justa Neves & Caldas, 2015). Given PEOs’ non-authoritative role in product classification, it is rather evident that it is crucial to deploy regulatory officers who are responsible for product classification to be assigned at entry points to address this challenge.

The issues of imported pharmaceutical products bearing inadequate or foreign language labels, as identified in this study, were similarly observed during import blitz examinations reported in the United States, illustrating the pervasive nature of this issue within pharmaceutical importations (Bro, 2005). Notably, the existing Malaysian regulations do not mandatorily require all imported drugs to bear complete labelling, provided the distributors fulfil labelling requisites before distributing them for commercial sale. Mandatory labelling is only stipulated for products containing poisons imported for personal use, as outlined in Regulation 4 of the Poisons Regulations 1952. Therefore, the current standard procedure upon encountering improperly labelled imported products by PEOs involves refusing entry of such products at borders. Initiation of investigation and prosecution by PEOs typically reserved for other types of offences or violations. The rationale for this practice, where no further action is taken against importers, is tied to considerations of cost and resource allocation, although this aspect may not have been extensively discussed in existing literature.

Fabricated identities, forged customs declarations and falsified documents are tactics used by unscrupulous importers to exploit the transparency gaps during customs clearance. These gaps are, in part, attributed to the absence of a web-based customs system that enable importers to electronically submit supporting documents for evaluation. The existing practice of manual submission and subsequent scrutiny of documents is susceptible to oversights and unethical practices such as corruption. Corruption has been perceived as one of the methods to introduce unregistered products into Malaysia(Nur Wahida Zulkifli et al., 2016b). Beyond economic implications, corruption may tarnish the reputation and credibility of enforcement agencies and pose a threat to public health (Sorato et al., 2024). Tackling this issue necessitates establishment of a more transparent customs system to ensure the integrity of pharmaceutical imports and to impede importation of unregistered products.

The absence of comprehensive legislation exacerbates the difficulties in effectively controlling sheer volume of websites, e-marketplaces and social media platforms that engage in offering unregistered pharmaceutical products. Technically, PEOs lack the authority to block websites, relying instead on their own initiatives to report such websites and listings to the MCMC and relevant online platforms. However, even MCMC doesn’t possess the direct authority to block websites under the Communications and Multimedia Act 1998, as Section 263(2) of the Act only states that Internet Service Providers must provide assistance in preventing violation of any Malaysian laws upon request by the authorities. In this context, endowing PED with statutory-based authority to proactively obstruct access to illicit websites would serve as a pragmatic measure. This move would reinforce the ongoing website surveillance activities undertaken by PEOs based on initiatives.

The persistent demand for unregistered pharmaceutical products from abroad continues to fuel their importation and consequently facilitates the presence and illicit trade of such products in the Malaysian market. One contributing factor is the perception among certain Malaysian consumers that overseas products, particularly those from developed countries, are of superior quality, leading to predisposition towards their consumption. This phenomenon finds support in a study by Mahmoud Sa'di Al-Haddad (2012), revealing a prevailing public inclination towards medications manufactured in developed countries due to the perceived higher standards of quality.

In addition to this quality-driven demand, a parallel economic-driven demand exists, particularly among immigrant populations in Malaysia (Ong et al., 2020). Unlike Malaysian citizens, immigrants are not generally entitled to subsidised public healthcare services, prompting them to seek more affordable alternatives. Consequently, the influx of unregistered products is further compounded by the cost advantage offered by selected foreign pharmaceutical products *vis-à-vis* local brick-and-mortar pharmacies. For instance, India, renowned for its production of low-cost pharmaceutical products and has emerged as a major supplier for illegal online pharmacies (Bate, 2013), is often the preferred source among immigrant communities.

The ongoing challenge of countering the demand for unregistered products signifies a significant shortfall in our current awareness programmes. This aligns with findings by Por et al. (2020), who reported that the majority of respondents in Kuala Lumpur, the capital city of Malaysia, demonstrated only moderately poor knowledge regarding counterfeit and adulterated pharmaceutical products. These insights collectively underscore the urgent need for more robust public education campaigns to raise awareness of the risks associated with unregistered pharmaceutical products and to reduce their demand across diverse segments of the population.

Building upon the discussion earlier, Table 3 delineates recommendations that are classified into three principal domains: legislative, administrative, and regulatory measures. These recommendations aim to address the issues and challenges encountered by enforcement officers effectively.

**Table 3.** Summary of recommendations: legislative, administrative, and regulatory measures.

**Measure**s****	**Strategies**
**Legislative Measures**	**I). Amendment of the** **Sale of Drugs Act 1952** **to endow PEOs with robust enforcement and investigative powers**
	(a) Power to examine packages. Specific provisions granting PEOs the power to halt, search or detain vehicles suspected of conveying unregistered products, and power to open and examine packages would alleviate the authority constraints at entry points.
	(b) Power to summon and examine witnesses. The power to order the attendance of witnesses through written notices to furnish documentation, information, or oral statements would facilitate investigation and mitigate the challenge of locating witnesses.
	(c) Power to compound offences. A provision authorising compounding of offence would enable PEOs to impose compounds to penalise illegal importers while expediting investigation. Obligating identified importers to make financial payments in addition to the forfeiture of their products at entry points would reduce recidivism and serve as a deterrence.
	(d) Insert the definition of ‘import'. Definition of ‘import' would enhance legislative clarity and distinguishes it from transit and transshipment activities.
	**II).** **Enactment of a new legislation that explicitly regulates online pharmacies and addresses internet-based pharmaceutical crimes.** Part IV of the Model Law on Medicine Crime proposed by Attaran (2015) serves as a comprehensive reference for outlining provisions related to the regulation of internet pharmacies (Nur Wahida et al, 2016b). Features that should be adopted involves:
	(a) Penalisation of internet domain name registrars who knowingly support illegal operations. ,
	(b) Power to block websites access. Conferring authority upon PEOs to issue direct instructions to Internet Service Providers or domain registrars for prompt removal of websites associated with unregistered products would expedite the existing procedures facilitated through MCMC or MYNIC.
**Administrative Measures**	**I). Fostering collaboration between enforcement agencies**
	(a) Routine joint operations at both land and water borders.
	(b) Establish dialogues to exchange expertise and synchronise operational protocols in bolstering cohesiveness between agencies.
	(c) Establish formal inter-agency task forces responsible for developing and implementing standardised communication channels and joint Standard Operating Procedures.
	**II). Expertise and resource allocation at entry points**
	(a) Deploy NPRA officers to provide immediate consultation to Custom Officers at entry points to ensure accurate product classification and facilitate goods referral to appropriate agencies for inspection.
	(b) Acquisition of additional scanners at all entry points, equipped with enhanced imaging capabilities capable of producing detailed 3D images of package contents. These scanners should be integrated with automated alert systems and advanced threat recognition software designed to detect anomalies typically linked to unregistered or counterfeit pharmaceutical products, such as irregular packaging, labelling inconsistencies, and concealed compartments.
	**III).** **Education and training programmes for PEO**s
	(a) In-depth training on the Malaysian pharmaceutical regulatory framework, including all pertinent legislation and guidelines issued by regulatory authorities.
	(b) Specialised workshops on counterfeit detection techniques such as recognising counterfeit packaging, identifying labeling discrepancies and verifying the authenticity of security Hologram label using authentication devices.
	(c) Training modules offered by organisations such as the International IP Crime Investigators College (IIPCIC) to stay abreast of global best practices in combating pharmaceutical crimes.
	(d) Courses on investigation and prosecution protocols to equip PEOs with necessary knowledge and skills such as investigative techniques, evidence collection and preservation, digital forensics, and legal procedures related to pharmaceutical crimes.
	**IV). Awareness programmes**
	(a) Collaborate with courier and shipping companies in educating their users about pharmaceutical import restrictions.
	(b) Forge collaboration with airlines to incorporate information onto their websites and strategic partnerships with the Ministry of Tourism to facilitate dissemination of importation restrictions to travellers intending to visit our country.
	(c) Implementation of more targeted public awareness initiatives through social media platforms to educate consumers on the risks associated with purchasing medicines from unauthorised online sources. Existing campaigns, such as the ‘Say No to Illegal Medicines' initiative launched by the Ministry of Health in 2024, should be sustained and expanded to maximise their reach and impact.
	**V). Enhancing transparency during customs clearance**
	Integrate blockchain technology within the customs system to provide customs officials with transparent access to essential documents and to reduce the risk of document forgery.
**Regulatory Measures**	**I). Enforcement against malpractice associated with importation**
	(a) Impose strict penalties to courier agents, shipping companies and importers who are found guilty of making incorrect declarations and falsifying documents.
	(b) Enforce suspension or revocation of operating licences for courier agents, shipping companies, and importers involved in such malpractice to deter future violations.
	(c) Intensify the frequency of random audits and inspections at entry points using risk-based profiling.

## 5. Conclusion

Despite the presence of legal and regulatory frameworks aimed at controlling importation of unregistered products, it is imperative that a comprehensive examination and review of our legal and regulatory framework be undertaken to adapt to evolving importation practices. The findings in this study revealed that the issues and challenges faced by enforcement officers in combating importation of unregistered products stem from inadequacies in the existing laws and practices. Curbing importation of unregistered pharmaceutical products requires multifaceted approaches that encompass legislative reforms, administrative transformation, rigorous enforcement, and robust collaboration among regulatory authorities and stakeholders. By diligently undertaking these measures, we can strive towards a more effective control over illicit importation of unregistered products, thereby safeguarding the well-being of the public.
